# Added value of diffusion weighted imaging in pediatric central nervous system embryonal tumors surveillance

**DOI:** 10.18632/oncotarget.19553

**Published:** 2017-07-25

**Authors:** Giovanni Morana, Cesar Augusto Alves, Domenico Tortora, Mariasavina Severino, Paolo Nozza, Armando Cama, Marcello Ravegnani, Gabriella D'Apolito, Alessandro Raso, Claudia Milanaccio, Claudia da Costa Leite, Maria Luisa Garrè, Andrea Rossi

**Affiliations:** ^1^ Neuroradiology Unit, Istituto Giannina Gaslini, Genova, Italy; ^2^ Radiology Institute, Hospital das Clinicas, Sao Paulo, Brazil; ^3^ Pathology Unit, Istituto Giannina Gaslini, Genova, Italy; ^4^ Neurosurgery Unit, Istituto Giannina Gaslini, Genova, Italy; ^5^ Neuroradiology Unit, Ospedale M. Bufalini, Cesena, Italy; ^6^ Neuro-Oncology Unit, Istituto Giannina Gaslini, Genova, Italy

**Keywords:** DWI, CNS embryonal tumors, medulloblastoma, ATRT, relapse

## Abstract

Diffusion weighted imaging (DWI) has an established role in primary CNS embryonal tumor (ET) characterization; however, its diagnostic utility in detecting relapse has never been determined. We aimed to compare DWI and conventional MRI sensitivity in CNS ET recurrence detection, and to evaluate the DWI properties of contrast-enhancing radiation induced lesions (RIL).

Fifty-six patients with CNS ET (25 with disease relapse, 6 with RIL and 25 with neither disease relapse nor RIL) were retrospectively evaluated with DWI, conventional MRI (including both T2/FLAIR and post-contrast images), or contrast-enhanced MR imaging (CE-MRI) alone. MRI studies were independently reviewed by two neuroradiologists for detection and localization of potential brain relapses. Sensitivity for focal relapse detection was calculated for each image set on a lesion-by-lesion basis. A descriptive per subject analysis was also performed. Evaluation of follow-up MRI studies served as standard of reference.

Focal recurrence detection sensitivity of DWI (96%) was significantly higher than conventional MRI (77%) and CE-MRI alone (51%) (p=0.0003 and p<0.0001). On per subject analysis there were not missed diagnoses for DWI. At the time of DWI relapse detection, conventional MRI missed 2 diagnoses, and CE-MRI 8. Analysis of medulloblastoma relapses revealed that DWI identified a higher number of focal lesions than CE-MRI in subjects with classic variant. All but one RIL did not show restricted diffusion.

In conclusion, DWI is a valuable complementary technique allowing for improved detection of focal relapse in CNS ET patients, particularly in children with classic medulloblastoma, and may assist in differentiating recurrence from RIL.

## INTRODUCTION

Central nervous system (CNS) embryonal tumors (ET) comprise a biologically heterogeneous group of malignant neoplasms occurring predominantly in children, generally composed of densely packed poorly differentiated cells (small blue cell tumors) with scant cytoplasm and high mitotic index [1]. According to the revised fourth edition of the WHO classification [2], the most common entities in this group include medulloblastomas (MB) (genetically or histologically defined), atypical teratoid/rhabdoid tumors (AT/RT), embryonal tumors with multilayered rosettes (ETMR), C19MC-altered and CNS embryonal tumors, NOS (replacing the CNS primitive neuroectodermal tumors of the fourth edition of the WHO classification).

ET demonstrate an increased tendency to recur and disseminate throughout the nervous system via cerebrospinal fluid pathways. Disease may recur at the primary site or may be disseminated at the time of relapse. Sites of distant relapse include the spinal leptomeninges and intracranial sites, in isolation or in any combination [3–5].

Contrast-enhanced MRI is the recommended standard to assess tumor spread and recurrence on imaging [6]. However, while the majority of primary CNS ET typically show contrast enhancement, the degree of enhancement can be variable, and considering only MB, approximately 11% to 25% do not enhance [7–10]. In addition, although MRI findings of pia-arachnoidal or focal nodular brain enhancement were previously described as highly specific in the diagnosis of recurrent disease [11], contrast enhancement may occur as a result of radiation therapy [12, 13], thus posing a major diagnostic challenge.

Diffusion weighted imaging (DWI) provides information regarding diffusion of water molecules in the section studied, from which quantitative values, the so-called apparent diffusion coefficient (ADC), can be calculated. DWI provides non-invasive estimation of differences in cell density and tissue structure and has been widely used in the diagnosis of pediatric brain tumors [14]. In particular, it has been demonstrated that CNS ET usually show decreased diffusivity in keeping with their common cellular features [9, 15, 16]. Nevertheless, even though the role of DWI in primary CNS ET characterization is well known, the diagnostic utility of DWI in detecting CNS ET relapse has not been conclusively established to our knowledge.

On the basis of these considerations, the aim of this study was to assess and compare DWI and conventional MRI sensitivity in CNS ET recurrence detection, and to evaluate the DWI properties of contrast-enhancing radiation induced lesions (RIL).

## RESULTS

Among 56 subjects enrolled in the study, 25 with disease relapse and 31 without relapse (6 with RIL and 25 without RIL) (Table 1), a total of 125 focal brain lesions were identified according to our standard of reference. In detail, there were 103 focal brain relapses and 22 radiation induced lesions. Nine subjects with focal disease relapse presented also with diffuse linear secondary pathological involvement. Isolated local relapse occurred in 4 patients (1 MB, 1 ETMR and 2 ATRT), combined local and distant relapse in 6 (all patients with MB) and isolated distant relapse in 15 (9 MB, 2 CNS ET, NOS, 4 ATRT). Distribution of focal relapsing lesions is reported in Table 2. Distribution of focal radiation-induced lesions was as follows: 13 local (11 cerebellar hemispheres, 2 middle cerebellar peduncles) and 9 distant (4 ependymal supratentorial, 5 thalami).

**Table 1:** Summary of subjects characteristics

No. of subjects			56		
Sex					
Male			35 (62.5%)		
Female			21 (37.5%)		
Age at diagnosis					
Median			5.4		
Range			0-14.3		
No. of subjects with relapse			25		
Histology					
MB	16	LCA 6		Group 3	2
				Group 4	1
				SHH	2
				Not available	1
		Classic 9		Group 3	2
				Group 4	2
				WNT	1
				Not available	4
		Desm/Nod 1		SHH	1
ATRT		6 (4 supratentorial, 2 infratentorial)			
CNS ET, NOS		2 (supratentorial)			
ETMR		1 (supratentorial)			
No. of subjects with RIL		6			
Histology					
MB			6	LCA 2	
			Classic 4		
No. of subjects with neither relapse nor RIL			25		
Histology					
			LCA 4		
MB	19		Classic 9		
			Desm/Nod 6		
CNS ET, NOS		4 (supratentorial)			
ATRT		2 (1 supratentorial, 1 infratentorial)			
Treatment					
CSI + Chemo			47 (84%)		
Chemo only			9 (16%)		
Time to recurrence (months)					
Median			18		
Range			4,8-118		
Time to RIL (months since the end of RT)					
Median			7,5		
Range			5-10		
Site of recurrence					
Local			4		
Distant			15		
Combined			6		
Pattern of recurrence					
Focal nodular			16		
Focal nodular and diffuse linear			9		
Survival in pts with recurrence					
Dead			21		
Alive			4		

MB: medulloblastoma, ATRT: atypical teratoid rhabdoid tumor, CNS ET, NOS: central nervous system embryonal tumor, not otherwise specified, ETMR: embryonal tumor with multi-layered rosette, LCA: large cell anaplastic, Desm: desmoplastic, RIL: radiation induced lesions, CSI: craniospinal irradiation.

**Table 2:** Distribution of focal relapsing lesions

		TOTALN patients 25 /N lesions 103	MBN patients 16 /N lesions 94	ATRTN patients 6 / N lesions 6	CNS ET, NOSN patients 2 / N lesions 2	ETMRN patients 1 / N lesions 1
Local		4/4	1/1	2/2	/	1/1
Distant		15/61	9/55	4/4	2/2	/
	Leptomeningeal	14/53	8/47	4/4	2/2	/
	Leptomeningeal and ependymal	1/8 (2 leptomeningeal 6 ependymal)	1/8 (2 leptomeningeal 6 ependymal)	/	/	/
Combined		6/38	6/38	/	/	/
	Local and ependymal	2/10 (2 local, 8 ependymal)	2/10 (2 local, 8 ependymal)	/	/	/
	Local and parenchymal	1/2 (1 local, 1 parenchymal)	1/2 (1 local, 1 parenchymal)	/	/	/
	Local and leptomeningeal	3/26 (3 local, 23 leptomeningeal)	3/26 (3 local, 23 leptomeningeal)	/	/	/

MB: medulloblastoma, ATRT: atypical teratoid rhabdoid tumor, CNS ET, NOS: central nervous system embryonal tumor, not otherwise specified, ETMR: embryonal tumor with multi-layered rosette.

Time to relapse ranged between 4.8 and 118 months after disease onset (median 18 months). Time to RIL onset ranged from 5 to 10 months (median 7.5 months) after the end of treatment.

**Figure 1:**
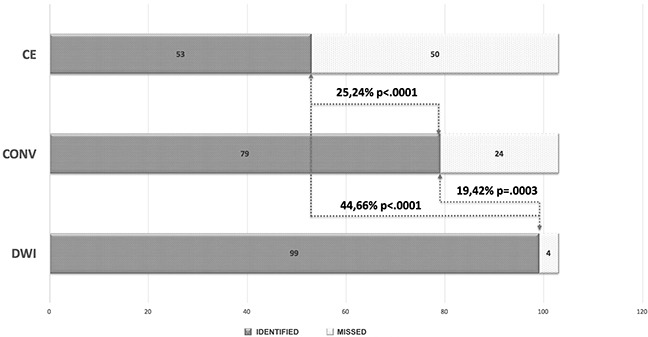
Bar chart showing identified (grey) and missed (dotted white) focal relapsing lesions for contrast enhanced (CE) MRI alone, conventional MRI (T2/FLAIR and post-contrast imaging) (CONV), and diffusion weighted imaging (DWI). DWI identified 19.42% more focal relapses than conventional MRI and 44.66% more lesions than CE-MRI alone. Conventional MRI identified 25.24% more focal relapses than CE-MRI.

Of 103 focal relapses, DWI correctly detected 99 lesions (96.1%), conventional MRI 79 (76.6%) and contrast-enhanced (CE)-MRI alone 53 (51.4%) at the blinded reading session. DWI identified 19.42% more focal relapses than conventional MRI (95% CI, 18.56-25.24, p=0.0003) and 44.66% more lesions than CE-MRI (95% CI, 9.42-24.99, p<0.0001). Conventional MRI identified 25.24% more focal relapses than CE-MRI (95% CI, 18.56-25.24, p<0.0001) (Figure 1).

Of 22 focal radiation induced contrast enhancing lesions, only 1 demonstrated decreased diffusion whereas the remainder showed increased diffusivity or diffusivity similar to normal parenchyma.

Interobserver agreement was excellent for every image set: DWI (ICC, 0.986; 95% CI, 0.972-0.993), conventional MRI (ICC, 0.989; 95% CI, 0.978-0.995) and CE-MRI (ICC, 0.994; 95% CI, 0.988-0.997).

A statistically significant difference was demonstrated between sensitivity of DWI (96%), conventional MRI (77%) and contrast enhanced (CE)-MRI (51%) (p=0.0003 and p<0.0001, respectively). A statistically significant difference was also demonstrated between conventional MRI and CE-MRI (p<0.0001). The positive predictive value (PPV) of DWI was 99%.

The average minimum and mean ADC values across relapsing lesions were 0.540 × 10^−3^ mm^2^/s ± 0.128 (SD) and 0.642 × 10^−3^ mm^2^/s ± 0.145 (SD), respectively. The average minimum and mean ADC values across RIL were 1.086 × 10^−3^ mm^2^/s ± 0.698 (SD) and 1.201 × 10^−3^ mm^2^/s ± 0.751 (SD). A significant difference was demonstrated between minimum and mean ADC of relapsing lesions and RIL (p<0.001 and p=0.001, respectively). The average minimum and mean ADC values across primary lesions were respectively 0.515 × 10^−3^ mm^2^/s ± 0.063 (SD) and 0.676 × 10^−3^ mm^2^/s ± 0.090 (SD). Evaluation of the contrast enhancement pattern of primary lesions revealed 10 non-enhancing tumors: 7 MB (6 classic, 1 anaplastic), 2 AT/RT and 1 CNS ET, NOS.

On per subject analysis, focal relapse was solely identified by DWI in two patients (8%) (1 MB, and 1 CNS ET, NOS). In 6 patients (24%) (all with MB), DWI identified a higher number of focal relapses when compared to conventional MRI. In 5 patients (20%) (2 MB, 1 ETMR, 2 ATRT), focal relapses were equally identified by DWI and conventional MRI, but lesions were non-enhancing on post-contrast imaging, thus in 13 out of 25 subjects with relapse (52%) DWI identified more focal lesions than CE-MRI alone. In 2 patients (2 MB), however, CE-MRI identified more focal relapses than DWI (8%). Finally, in 10 patients (40%) (5 MB, 1 CNS ET, NOS, and 4 ATRT) all imaging modalities identified the same number of lesions. Overall, there were no missed diagnoses for DWI, whereas conventional MRI missed 2, and CE-MRI missed 8 diagnoses at the time of DWI relapse detection. Lack of enhancement of relapsing lesions was demonstrated in 4 out of 16 patients with MB (3 classic and 1 large-cell/anaplastic), in the patient with ETMR, in 1 out of 2 patients with CNS ET, NOS and in 2 out of 6 patients with AT/RT.

When only considering MB, DWI identified a higher number of focal relapses than CE-MRI alone in 9 out of 16 subjects (56%). Of these, histology of primary lesions consisted of 7 classic, 1 desmoplastic, and 1 large-cell/anaplastic, whereas molecular subgrouping revealed two group 4, two SHH, one group 3, and one WNT (molecular subgrouping unavailable in 3 subjects). In those subjects (7 out of 16) in whom CE-MRI identified an equal (5 subjects) or higher (2 subjects) number of focal lesions than DWI, histology revealed 5 large-cell/anaplastic and 2 classic MB (three group 3, one SHH and one group 4; molecular subgrouping unavailable in 2 subjects). A significant difference was demonstrated between histological variant distribution and detection rate of DWI and CE-MRI images (p=0.043).

Regarding diffuse linear leptomeningeal or ependymal relapse, CE-MRI was positive in 7 out of 9 patients (78%) (4 MB and 3 ATRT) whereas DWI in 3 out of 9 patients (33%) (1 MB, 1 CNS ET, NOS and 1 ATRT). Of note, in 2 patients (1 MB and 1 CNS ET, NOS) CE-MRI was negative at the time of DWI diffuse linear relapse detection.

**Figure 2:**
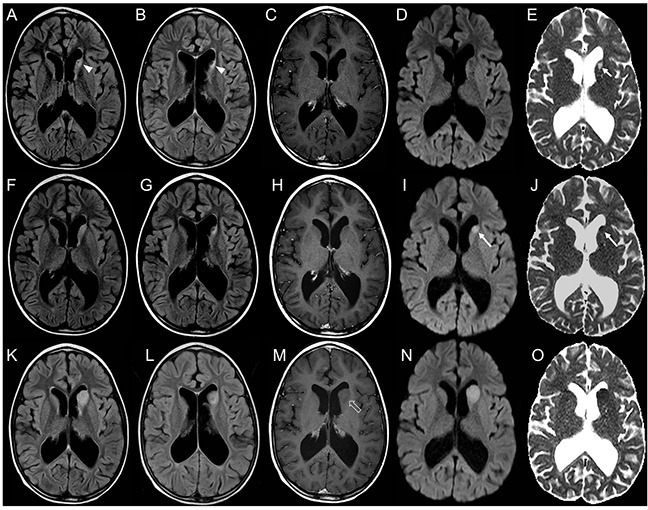
Brain MRI findings in an 8-year-old boy with focal distant medulloblastoma relapse detected earlier by DWI Axial FLAIR **(A, B)** and post-contrast T1-weighted **(C)** images reveal a small focal gliotic area in the head of the left caudate nucleus (arrowheads, A, B). DWI **(D)** and corresponding ADC **(E)** map show facilitated diffusion (short thin arrow, E). This sequela was stable for 2 years without evidence of disease relapse. Subsequent brain MRI **(F-J)** does not reveal significant changes when compared to the prior study on both FLAIR **(F, G)** and post-contrast T1-weighted images **(H)**. At that time conventional pre- and post-contrast MRI were considered negative for relapse. On the contrary DWI **(I)** and ADC **(J)** map clearly show a change in signal intensity of the left caudate head nucleus focal lesion, demonstrating decreased diffusivity (long thin arrows, I, J); evaluation of DWI/ADC images was considered positive for relapse. Follow-up MRI **(K-O)** shows increased volume of the focal lesion, with persistent lack of contrast enhancement (open arrow, M) and decreased diffusivity **(N, O)**, confirming focal relapse.

**Figure 3:**
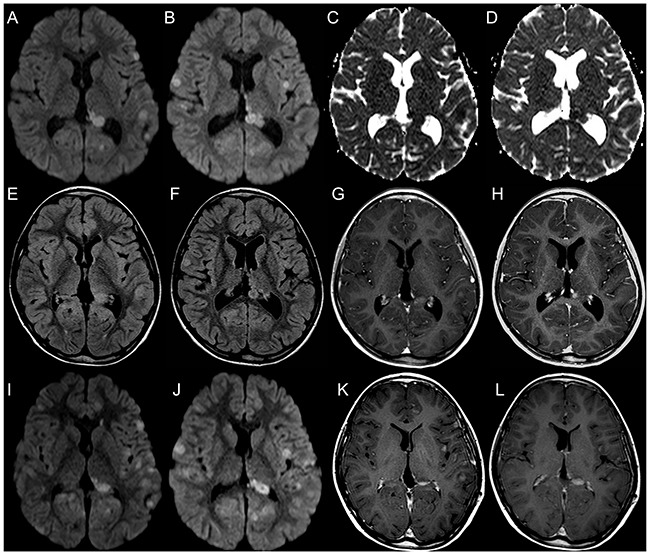
Brain MRI findings in a 12-year-old girl with multiple focal distant medulloblastoma relapsing lesions better depicted by DWI DWI images **(A, B)** and corresponding ADC maps **(C, D)** reveal multiple leptomeningeal and ependymal focal nodular lesions with decreased diffusivity. FLAIR **(E, F)** images show a lower number of lesions, and only few of them demonstrate contrast enhancement **(G, H)**. On follow-up MRI, DWI **(I, J)** and post-contrast T1-weighted images **(K, L)** demonstrate increased number and volume of focal lesions, confirming multifocal disease relapse.

**Figure 4:**
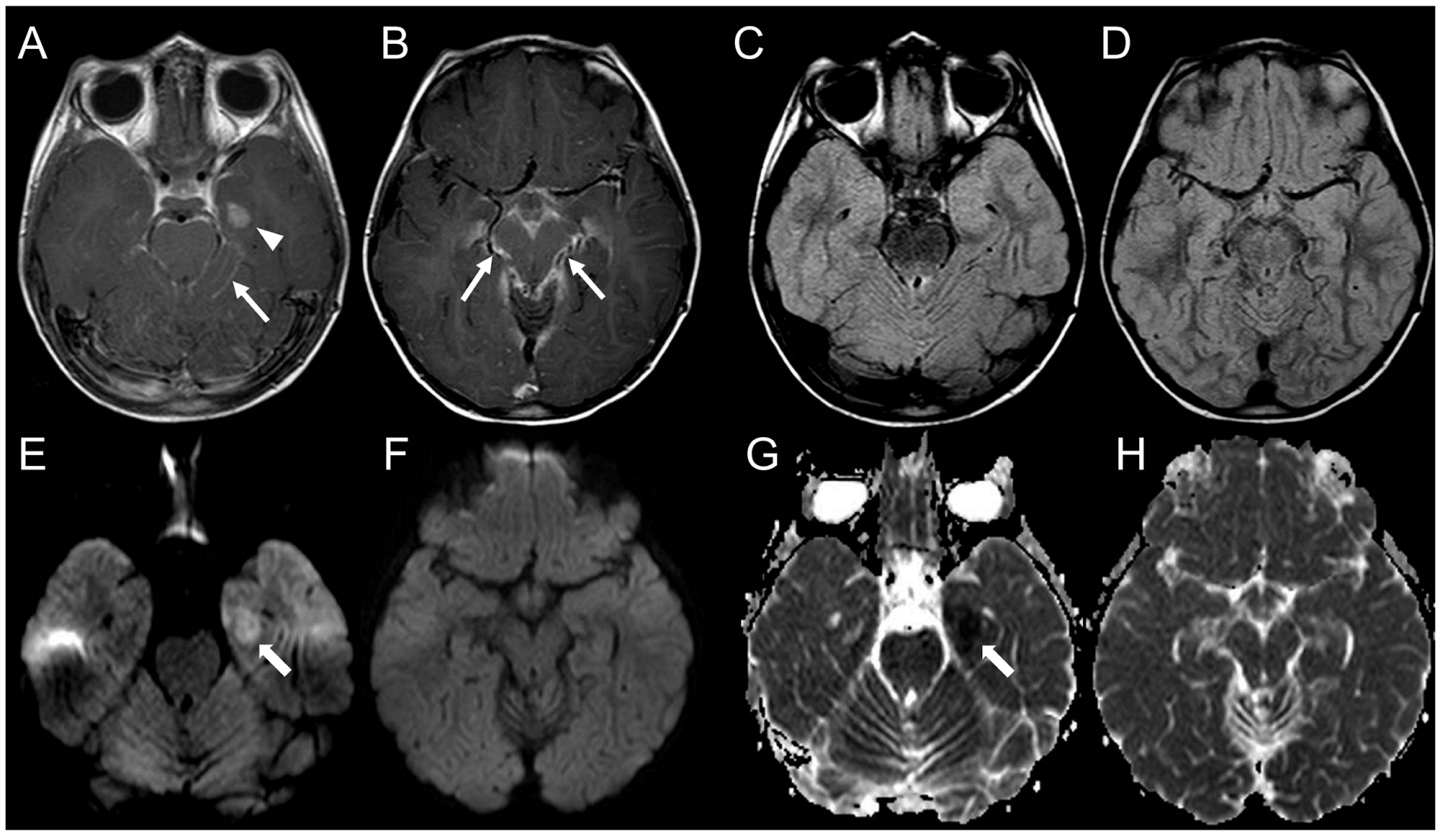
Brain MRI findings in a 7-year-old girl with AT/RT relapse and linear leptomeningeal involvement not visible on DWI Post-contrast T1-weighted images show a distant focal nodular contrast enhancing relapsing lesion in the left temporal lobe (arrowhead, **A**); there is also diffuse linear leptomeningeal enhancement along cerebellar folia and in the basal cistern (long thin arrows, **A, B**). FLAIR images do not reveal evident lesions **(C, D)**. DWI and corresponding ADC maps clearly show the nodular lesion with decreased diffusivity in the left temporal lobe (thick arrows, **E, G**) but are unable to demonstrate linear leptomeningeal involvement **(F, H)**.

**Figure 5:**
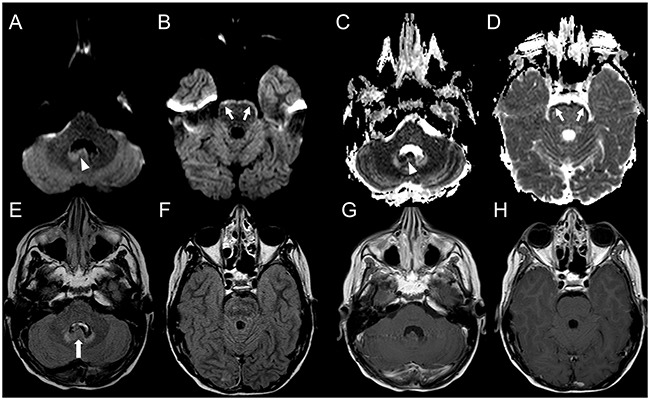
Brain MRI findings in a 12-year-old boy with CNS ET, NOS relapse and linear leptomeningeal involvement visible only on DWI DWI **(A, B)** and corresponding ADC **(C, D)** maps show two adjacent focal nodular ependymal relapsing lesions with decreased diffusivity located in the right posterolateral recess of the IV ventricle and along the nodule of vermis (arrowheads, A, C). There is also linear decreased diffusivity along the ventral margin of the brainstem in keeping with linear leptomeningeal involvement (short thin arrows, B, D). FLAIR images **(E, F)** show a reduced representation of the right posterolateral recess of the IV ventricle and focal prominence of the nodule of vermis (thick arrow, E). Post-contrast T1-weighted images **(G, H)** do not reveal enhancing lesions.

**Figure 6:**
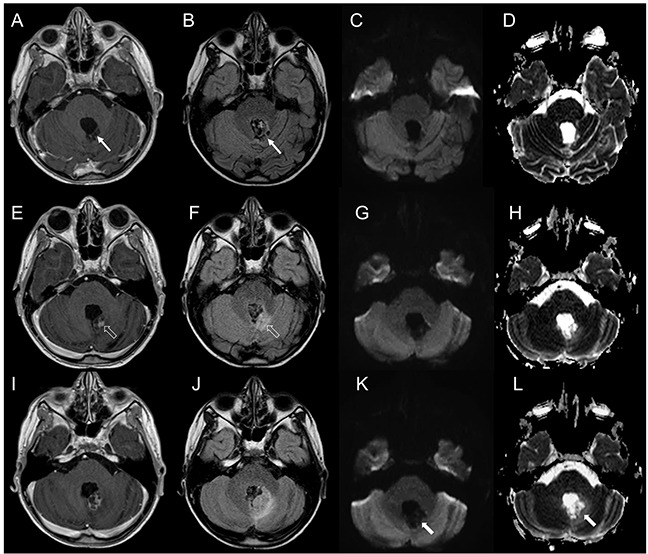
Brain MRI findings in a patient with non-neoplastic radiation induced focal lesion **(A-D)**. Post-operative MRI 3 months following surgery. Post-contrast T1-weighted (A) and FLAIR (B) images demonstrate a post-surgical cavity along the left posterolateral margin of the fourth ventricle without evidence of residual/recurrent disease (thin arrows, A, B). DWI (C) and corresponding ADC map (D) show increased diffusion. **(E-H)** Follow-up MRI 5 months after the end of combined CT and RT. Brain MRI shows a new area of contrast-enhancement (E) with minimal mass effect and FLAIR hypersignal (F) along the posterolateral margin of the fourth ventricle (open arrows, E, F). DWI (G) and corresponding ADC map (H) show increased diffusion. **(I-L)** Follow-up MRI 7 months after the end of combined CT and RT. Post-contrast T1-weighted (I) and FLAIR (J) images show increased extension of the lesion with perilesional edema. On DWI and ADC there still is facilitated diffusion (thick arrows, K, L). The lesion was surgically removed and histology revealed gliosis and hemosiderin-laden macrophages in keeping with therapy-induced reactive changes.

Representative images of relapsing and radiation induced lesions are shown in Figures 2–6.

## DISCUSSION

Standard surveillance for CNS ET includes neuroimaging and clinical evaluations at regular intervals. The rationale behind surveillance imaging is that early detection of tumor recurrence may potentially allow for prompt intervention and thus improved outcome. However, screening for and diagnosing recurrent disease poses significant challenges. Early identification of tumor relapse can be influenced by the contrast-enhancing pattern of the lesion. Of note, not all CNS ET present with contrast enhancement, and prior studies have demonstrated that metastases of MB tend to display a weaker enhancement than the initial neoplasms [17]; in addition, up to 36% of MB recurrences can be non-enhancing [10] with up to 20% of secondary leptomeningeal diseases missed by conventional MRI [18]. Recent studies have also demonstrated that depending on the molecular subgroup, MBs can present with higher or lower degrees of blood brain barrier breakdown [19]. For instance, lack of enhancement has been demonstrated in a significant subset of group IV MB [9]. AT/RT and others CNS ET can also be non-enhancing.

At the same time, on conventional MRI, it may be difficult to differentiate tumor recurrence from other entities, such as radiation-induced contrast-enhancement, and misdiagnosing recurrent lesions can have a significant impact on patient's outcome. Despite these shortcomings, conventional post-contrast MRI is presently considered the standard of reference for evaluating recurrent disease on neuroimaging.

The rationale of this work was based on the known evidence of decreased diffusivity of these tumors, so far reported in the vast majority of cases [8, 9, 15, 20, 21]. Increased cellularity, decreased extracellular space, and high nuclear-to-cytoplasmic ratio are the most common explanation of the DWI features of CNS ET. Isolated reports or small case series have also suggested a potential role of DWI in MB surveillance. Shubert et al. [22] evaluated the diagnostic utility of DWI in 3 patients with MBs suggesting a role for DWI in early detection of metastatic disease and warranting a formal study. Similar observations were reported by Rasalkar et al. [23] and by Amene et al. [24] showing in isolated case reports the utility of DWI in detecting recurrent MBs. Nevertheless, with the exception of the sparse above-mentioned data, no systematic studies aimed at evaluating the diagnostic value of DWI in the surveillance of children with CNS embryonal tumors are as yet available to our knowledge.

Our results show that DWI was able to pick up a significantly higher number of focal CNS ET relapses when compared to conventional MRI. Sensitivity for focal relapsing lesions raised from 77% of conventional MRI to 96% of DWI (p=0.0003). When considering a per subject analysis, DWI added diagnostic information to conventional MRI in 32% of patients with focal nodular disease relapse, being the sole diagnostic tool to show early tumor recurrence in two subjects (8%) and showing a higher number of lesions than CE-MRI in 13 out of 25 subjects (52%). At the same time, all but one lesion with decreased diffusivity turned out to be secondary lesions, yielding a very high PPV (99%). The high sensitivity and positive predictive value suggest that DWI is a good test in identifying focal brain CNS ET relapses, so that a positive finding on DWI is highly predictive of relapse.

Focal radiation induced contrast-enhancing lesions in the setting of CNS ET surveillance have been widely documented [13, 25–27] but almost all studies have focused on conventional MRI characteristics and temporal evolution of lesions, rather than on DWI. The most common pattern of RIL in children with CNS ET treated with concurrent radiotherapy and chemotherapy is characterized by small foci of contrast enhancement within the brain parenchyma, almost always associated with focal T2/FLAIR hyperintensity. These lesions are more frequent in children treated with hyperfractionated accelerated radiotherapy (HART) and are considered a milder form of brain injury due to transient vasogenic edema or demyelination with blood brain barrier (BBB) breakdown, different from classic radiation necrosis; they usually occur within the white matter at variable distances from the primary tumor and at variable intervals after the end of treatment, ranging from few months to years [13, 28]. On follow-up, they usually regress in size, although interval increase can be detected on initial evaluations, followed by subsequent decrease; progression to radiation necrosis should be suspected if they progressively increase in size or are accompanied by edema and mass effect [29]. Our findings show that all but one focal contrast-enhancing RIL did not present with decreased diffusion, suggesting that lack of decreased diffusion favors a non-tumoral lesion. Indeed, average minimum and mean ADC values of focal relapses and contrast-enhancing RIL demonstrated significant differences among the two groups. Our study also confirms that a distant intra-axial parenchymal relapse is extremely uncommon in CNS ET, as all but one distant focal parenchymal enhancing lesions turned out to be radiation induced lesions.

Regarding diffuse linear leptomeningeal involvement, our data confirm that post-contrast imaging is the mainstay to evaluate this pattern of relapse due to higher sensitivity. However, even on DWI linear leptomeningeal involvement can sometimes be appreciated resulting in a diffuse DWI highlighting of the pial surface of involved brain areas.

Analysis of relapsing MB demonstrated that in subjects with the histologically defined classic variant, DWI picked-up a higher number of focal lesions when compared to CE-MRI alone; classic MB relapsing lesions also presented lack of contrast enhancement with higher frequency. At the same time lack of enhancement of primary MB was documented mainly in classic variants (6 out of 7 non-enhancing tumors). Interestingly, a similar observation (regarding primary lesions) was reported by Fruehwald-Pallamar J et al. [30], who documented subtle or linear enhancement only in classic MB, whereas all other MB variants presented marked enhancement. A similar pattern can be inferred by the study of Yeom KW et al. [8] in which 8 out of 9 non-enhancing primary MB belonged to the classic group. In a more recent study by Łastowska M et al. [31] evaluating contrast enhancement pattern of patients with non-WNT/SHH MB, none to weak enhancement was demonstrated in 29 out of 60 subjects, of which 25 were classic MB. This aspect highlights the role of DWI in surveillance imaging of classic MB. Intriguingly, although MB does not change molecular subgroup at recurrence [32] and remains stable among primary and metastatic compartments [33], histological subtypes of MB may change upon recurrence [34]. However, as demonstrated by Pöschl J et al. [34]in a series of 16 relapsing medulloblastomas in which 7 out of 16 cases (44 %) showed change in the histological subtype, the majority of desmoplastic/nodular medulloblastoma cases (57 %) relapsed as classic medulloblastoma, whereas all classic medulloblastoma but one remained stable among primary and relapsing compartment. This observation may potentially give an explanation of the neuroimaging evidence of weaker contrast enhancement of relapsing MB lesions when compared with primary tumors, and may further strengthen the role of DWI in MB surveillance given the high frequency of tumors that relapse as classic variants which, as previously demonstrated, tend to be more frequently non-enhancing.

Our results should be interpreted with awareness of certain limitations. First, given the retrospective nature of the study, sampling bias cannot be excluded. We are also aware of the relatively small sample of patients; however, per lesion analysis allowed to evaluate a significant number of lesions. We did not evaluate post-contrast FLAIR imaging in our study since our routine Institutional brain tumor surveillance protocol does not include this sequence following gadolinium administration. We recognize that post-contrast FLAIR imaging can add significant information to conventional post-contrast T1-weighted imaging for the evaluation of leptomeningeal diseases, improving detection of leptomeningeal metastasis in children [35, 36]; further investigations aimed at comparing the diagnostic value of DWI and post-contrast FLAIR imaging in ET surveillance are awaited to specifically evaluate the contribution of these sequences.

Since molecular subgrouping of MBs was not available for all patients, we were unable to draw definite conclusion regarding possible correlations between DWI characteristics of relapsing lesions and molecular subgroups; further studies with larger cohorts are thus needed to evaluate whether surveillance imaging of particular subgroups of MB may be improved by careful evaluation of DWI.

With the exception of two subjects, histological confirmation of relapsing and radiation induced lesions was not obtained for both clinical and ethical reasons, and radiographic evaluation was the standard of reference; however, MRI represents a common standard in clinical practice and current standard of care does not require histological confirmation of relapsing lesions.

In conclusion, DWI represents a valuable diagnostic tool for the surveillance of embryonal tumors after treatment and can provide helpful information for clinical management of these patients. In particular, DWI may improve the detection of focal recurrent disease and assist in differentiating recurrent disease from contrast-enhancing radiation induced lesions. Careful evaluation of DWI images is therefore strongly recommended. Among children with MB, particular attention should be paid to subjects with classic variant who present a higher frequency of non-enhancing lesions, better evaluable with DWI.

## MATERIALS AND METHODS

This retrospective single-center study was institutional review board-approved. Informed consent was waived by the local institutional review board.

### Subjects

We screened the tumor registry and radiology report database of our Institution for patients with histologically confirmed CNS ET aged less than 18 years at initial diagnosis, and for whom MRI data including DWI at diagnosis and on serial follow-up were available in our Picture Archive and Communication System (PACS). Patients were categorized in three main groups: i) those with a brain disease relapse, ii) those without relapse, presenting with contrast-enhancing radiation-induced brain lesions, and iii) those without evidence of disease relapse or of radiation induced brain lesions. Specifically, patients with disease relapse were selected on the basis of a pathologic confirmation or radiologic determination (according to current standard radiological criteria) [5] and clinical evolution; subjects with contrast enhancing RIL were selected on the basis of a pathologic confirmation or after revision of the radiological reports, describing new onset of cerebral focal contrast enhancing lesions following radiotherapy, that demonstrated spontaneous regression on follow-up without treatment. Absence of brain RIL and disease relapse proved at MR imaging follow-up of at least 3 years was the selection criteria for the last group of subjects.

Fifty-six subjects (34 males and 22 females) were identified. There were 25 patients with disease relapse, and 31 without relapse (6 with RIL and 25 without RIL). Among 25 patients with disease relapse there were 16 MB, 1 ETMR (previously designated as embryonal tumor with abundant neuropil and true rosettes), 2 CNS embryonal tumors, NOS (previously designated as PNET), and 6 AT/RT. All patients with RIL had MB (5 out of 6 treated with Hyperfractionated Accelerated Radiotherapy). Among patients with neither disease relapse nor RIL there were 19 MB, 4 CNS embryonal tumors, NOS and 2 AT/RT. The main characteristics of subjects and their brain lesions are summarized in Table 1.

### MRI protocol

All MRI studies were performed on a 1.5 Tesla magnet (Intera Achieva; Philips, Best, the Netherlands). Conventional imaging included 4-mm-thick axial Fluid Attenuated Inversion Recovery (FLAIR), T2- and T1-weighted images. Following gadolinium compound bolus administration (0.1 mmol/kg, macrocyclic ionic agent), 4-mm-thick axial, coronal, and sagittal T1- weighted images were acquired in all patients.

DWI was performed using a single-shot spin-echo (SE) echo-planar sequence with the following parameters: TR/TE= 5.050/63 ms, 90° flip angle, NEX=2, 30 transverse sections, SENSE factor= 2.5, slice thickness/gap=4 mm/1mm, FOV= 160 mm, 100×160 matrix, and imaging time of 69 s. Diffusion sensitizing gradients were applied in the x, y, and z directions with b factors of 0 and 1000 s/mm^2^.

### Study design, image evaluation and analysis

Since the main objective of the study was to evaluate the recurrence detection rate of DWI in comparison with pre- and post-contrast MRI, an independent pre-evaluation of all MRIs performed during follow-up of each patient with documented disease relapse was carried out by the principal investigator (AR, 24 years of experience) in order to select the studies corresponding to radiographic relapse onset. Radiographic relapse was defined as new onset of local disease, brain metastasis, or both. In detail, considering the purpose of our analysis, studies were considered positive for tumor relapse in case of new onset of at least one of the following: i) focal nodular (minimum diameter on transverse plane of 3 mm) or diffuse linear intracranial contrast enhancement, ii) focal lesion on T2/FLAIR images, even without contrast enhancement, clearly distinguishable from comorbid events (eg, demyelination, ischemic injury, infection, bleeding, etc), iii) focal nodular or diffuse linear hyperintense signal relative to the surrounding normal brain on DWI and hypointense signal on the corresponding ADC map (decreased diffusion) [37], clearly distinguishable from artifactual images, ischemic or haemorrhagic lesions. When evaluating MRI scans, both conventional imaging and DWI were available to the principal investigator. Findings were compared with follow-up MRI studies and clinical data in order to establish and confirm the date of radiographic relapse.

Then, he evaluated the MRI scans of patients with focal contrast enhancing RIL and the examination date corresponding to RIL onset was recorded.

Finally, he reviewed all MRI studies of patients with absence of disease relapse and RIL and selected an intermediate follow-up study.

In a following image reading session, two different neuroradiologists with 12 and 3 years of experience, respectively (GM and CAA), were asked to independently review all the selected MRI studies (patients with and without relapse, including RIL). These observers were blinded to the final diagnosis. Three sets of images were analysed for each patient: conventional MRI (axial FLAIR, T1 and T2-weighted images) including axial contrast enhanced (CE) T1-weighted images (image set A), only axial CE T1-weighted images (image set B), and only DWI/ADC images (image set C). To minimize any recall bias, the readings of each image set were separated by 2 weeks and images were analysed in a random order. These observers were asked to determine potential tumor relapses utilizing the same criteria used by the principal investigator. Each image set was evaluated in comparison with the corresponding image set of the prior MRI study performed by each patient. For each image set the two readers independently recorded the number and location of potential tumor relapses (local or distant leptomeningeal, ependymal or parenchymal) along with the MRI pattern (focal nodular or diffuse linear). Confluent lesions were counted as one. Potential local relapse was defined as recurrence within the tumor primary area. Potential distant relapse was defined as recurrence outside the primary tumor area. The same 2 neuroradiologists jointly reviewed all the image sets with discordant evaluation. They were asked to reach a consensus on the number and location of lesions for each patient. This consensus reading was considered for statistical analysis.

After the blinded reading was completed, the DWI along with pre-and post-contrast MRI images were reviewed together by all three readers in consensus; they compared the results of the blinded reading with the standard of reference. During this final verification, region of-

interest-based ADC analysis of focal brain relapsing lesions and primary lesions was performed as previously described [8]. Minimum and mean ADC values of RIL were also calculated by manually drawing a region of interest (ROI) onto the obtained ADC maps in the area corresponding to the T1-enhancing region. ROIs were shaped to the contour of contrast enhancement.

Presence or absence of contrast-enhancement of primary lesions was finally evaluated.

### Standard of reference

Because histological proof was not available in all but one relapsing lesion and one RIL, evaluation of all combined image sets and follow-up MRIs were considered the image comparator. Any identified lesion increasing in size on follow-up serial MR examinations or decreasing following treatment was considered positive for relapse [38]. Enhancing lesions following radiotherapy that disappeared or decreased in subsequent MR examinations without any treatment were considered radiation injury related and negative for relapse [39].

### Statistical analysis

Statistical analysis was performed by using SPSS Statistics for Mac, Version 21.0 (IBM, Armonk, New York). A p value of.05 was used to define nominal statistical significance. Differences among the three image sets were evaluated on a per lesion and per subject analysis. Intraclass correlation coefficients (ICCs) were used to study the agreement between the 2 readers in identifying the number and location of potential relapsing lesions. An ICC < of 0.40 indicated a poor agreement; 0.40–0.75, fair-to-good (moderate); and 0.76–1.00, excellent agreement [40]. A McNemar test was used to evaluate the differences in detection score of relapsing lesions among the three image sets. Sensitivity (SE) for all image sets and positive predictive value (PPV) for DWI were calculated by comparing the blinded reading results with the standard of reference.

Recurrence and RIL groups were compared by using Mann-Whitney U test for average minimum and mean ADC values.

Chi-squared test was performed to assess differences between DWI and CE images focal relapse detection rates among MB variants.

## References

[1] PDQ Pediatric Treatment Editorial Board Childhood Central Nervous System Embryonal Tumors Treatment (PDQ®): Health Professional Version. PDQ Cancer Information Summaries.

[2] Louis DN, Perry A, Reifenberger G, von Deimling A, Figarella-Branger D, Cavenee WK, Ohgaki H, Wiestler OD, Kleihues P, Ellison DW (2016). The 2016 World Health Organization classification of tumors of the central nervous system: a summary. Acta Neuropathol.

[3] Perreault S, Lober RM, Carret AS, Zhang G, Hershon L, Décarie JC, Vogel H, Yeom KW, Fisher PG, Partap S (2014). Surveillance imaging in children with malignant CNS tumors: low yield of spine MRI. J Neurooncol.

[4] Warmuth-Metz M, Blashofer S, von Bueren AO, von Hoff K, Bison B, Pohl F, Kortmann RD, Pietsch T, Rutkowski S (2011). Recurrence in childhood medulloblastoma. J Neurooncol.

[5] Perreault S, Lober RM, Carret AS, Zhang G, Hershon L, Décarie JC, Yeom K, Vogel H, Fisher PG, Partap S (2013). Relapse patterns in pediatric embryonal central nervous system tumors. J Neurooncol.

[6] Koeller KK, Rushing EJ (2003). From the archives of the AFIP: medulloblastoma: a comprehensive review with radiologic-pathologic correlation. Radiographics.

[7] Erdem E, Zimmerman RA, Haselgrove JC, Bilaniuk LT, Hunter JV (2001). Diffusion-weighted imaging and fluid attenuated inversion recovery imaging in the evaluation of primitive neuroectodermal tumors. Neuroradiology.

[8] Yeom KW, Mobley BC, Lober RM, Andre JB, Partap S, Vogel H, Barnes PD (2013). Distinctive MRI features of pediatric medulloblastoma subtypes. Am J Roentgenol.

[9] Perreault S, Ramaswamy V, Achrol AS, Chao K, Liu TT, Shih D, Remke M, Schubert S, Bouffet E, Fisher PG, Partap S, Vogel H, Taylor MD (2014). MRI surrogates for molecular subgroups of medulloblastoma. AJNR Am J Neuroradiol.

[10] Rollins N, Mendelsohn D, Mulne A, Barton R, Diehl J, Reyes N, Sklar F (1990). Recurrent medulloblastoma: frequency of tumor enhancement on Gd-DTPA MR imaging. Am J Roentgenol.

[11] Meyers SP, Wildenhain S, Chess MA, Tarr RW (1994). Postoperative evaluation for intracranial recurrence of medulloblastoma: MR findings with gadopentetate dimeglumine. Am J Neuroradiol.

[12] Weintraub L, Miller T, Friedman I, Abbott R, Levy AS (2014). Misdiagnosing recurrent medulloblastoma: the danger of examination and imaging without histological confirmation. J Neurosurg Pediatr.

[13] Spreafico F, Gandola L, Marchianò A, Simonetti F, Poggi G, Adduci A, Clerici CA, Luksch R, Biassoni V, Meazza C, Catania S, Terenziani M, Musumeci R (2008). Brain magnetic resonance imaging after high-dose chemotherapy and radiotherapy for childhood brain tumors. Int J Radiat Oncol Biol Phys.

[14] Rossi A, Gandolfo C, Morana G, Severino M, Garrè ML, Cama A (2010). New MR sequences (diffusion, perfusion, spectroscopy) in brain tumours. Pediatr Radiol.

[15] Rumboldt Z, Camacho DL, Lake D, Welsh CT, Castillo M (2006). Apparent diffusion coefficients for differentiation of cerebellar tumors in children. Am J Neuroradiol.

[16] Koral K, Gargan L, Bowers DC, Gimi B, Timmons CF, Weprin B, Rollins NK (2008). Imaging characteristics of atypical teratoid-rhabdoid tumor in children compared with medulloblastoma. Am J Roentgenol.

[17] Bühring U, Strayle-Batra M, Freudenstein D, Scheel-Walter HG, Küker W (2002). MRI features of primary, secondary and metastatic medulloblastoma. Eur Radiol.

[18] Fouladi M, Gajjar A, Boyett JM, Walter AW, Thompson SJ, Merchant TE, Jenkins JJ, Langston JW, Liu A, Kun LE, Heideman RL (1999). Comparison of CSF cytology and spinal magnetic resonance imaging in the detection of leptomeningeal disease in pediatric medulloblastoma or primitive neuroectodermal tumor. J Clin Oncol.

[19] Guerit S, Liebner S (2016). Blood-brain barrier breakdown determines differential therapeutic outcome in genetically diverse forms of medulloblastoma. Cancer Cell.

[20] Jaremko JL, Jans LB, Coleman LT, Ditchfield MR (2010). Value and limitations of diffusion-weighted imaging in grading and diagnosis of pediatric posterior fossa tumors. Am J Neuroradiol.

[21] Chawla A, Emmanuel JV, Seow WT, Lou J, Teo HE, Lim CC (2007). Paediatric PNET: pre-surgical MRI features. Clin Radiol.

[22] Schubert MI, Wilke M, Müller-Weihrich S, Auer DP (2006). Diffusion-weighted magnetic resonance imaging of treatment-associated changes in recurrent and residual medulloblastoma: preliminary observations in three children. Acta Radiol.

[23] Rasalkar DD, Chu WC, Shing MK, Li CK (2011). Medulloblastoma with leptomeningeal metastases. Hong Kong Med J.

[24] Amene CS, Yeh-Nayre LA, Crawford JR (2013). Isolated sensorineural hearing loss as initial presentation of recurrent medulloblastoma: neuroimaging and audiologic correlates. Clin Neuroradiol.

[25] Helton KJ, Edwards M, Steen RG, Merchant TE, Sapp MV, Boop FA, Langston J (2005). Neuroimaging-detected late transient treatment-induced lesions in pediatric patients with brain tumors. J Neurosurg.

[26] Fouladi M, Chintagumpala M, Laningham FH, Ashley D, Kellie SJ, Langston JW, McCluggage CW, Woo S, Kocak M, Krull K, Kun LE, Mulhern RK, Gajjar A (2004). White matter lesions detected by magnetic resonance imaging after radiotherapy and high-dose chemotherapy in children with medulloblastoma or primitive neuroectodermal tumor. J Clin Oncol.

[27] Muscal JA, Jones JY, Paulino AC, Bertuch AA, Su J, Woo SY, Mahoney DH, Chintagumpala M (2009). Changes mimicking new leptomeningeal disease after intensity-modulated radiotherapy for medulloblastoma. Int J Radiat Oncol Biol Phys.

[28] Kumar AJ, Leeds NE, Fuller GN, Van Tassel P, Maor MH, Sawaya RE, Levin VA (2000). Malignant gliomas: MR imaging spectrum of radiation therapy–and chemotherapy-induced necrosis of the brain after treatment. Radiology.

[29] Pruzincová L, Steno J, Srbecký M, Kalina P, Rychlý B, Boljesíková E, Chorváth M, Novotný M, Procka V, Makaiová I, Belan V (2009). MR imaging of late radiation therapy- and chemotherapy-induced injury: a pictorial essay. Eur Radiol.

[30] Fruehwald-Pallamar J, Puchner SB, Rossi A, Garre ML, Cama A, Koelblinger C, Osborn AG, Thurnher MM (2011). Magnetic resonance imaging spectrum of medulloblastoma. Neuroradiology.

[31] Łastowska M, Jurkiewicz E, Trubicka J, Daszkiewicz P, Drogosiewicz M, Malczyk K, Grajkowska W, Matyja E, Cukrowska B, Pronicki M, Perek-Polnik M, Perek D, Dembowska-Bagińska B (2015). Contrast enhancement pattern predicts poor survival for patients with non-WNT/SHH medulloblastoma tumours. J Neurooncol.

[32] Ramaswamy V, Remke M, Bouffet E, Faria CC, Perreault S, Cho YJ, Shih DJ, Luu B, Dubuc AM, Northcott PA, Schüller U, Gururangan S, McLendon R (2013). Recurrence patterns across medulloblastoma subgroups: an integrated clinical and molecular analysis. Lancet Oncol.

[33] Wang X, Dubuc AM, Ramaswamy V, Mack S, Gendoo DM, Remke M, Wu X, Garzia L, Luu B, Cavalli F, Peacock J, López B, Skowron P (2015). Medulloblastoma subgroups remain stable across primary and metastatic compartments. Acta Neuropathol.

[34] Pöschl J, Koch A, Schüller U (2015). Histological subtype of medulloblastoma frequently changes upon recurrence. Acta Neuropathol.

[35] Ercan N, Gultekin S, Celik H, Tali TE, Oner YA, Erbas G (2004). Diagnostic value of contrast-enhanced fluid-attenuated inversion recovery MR imaging of intracranial metastases. Am J Neuroradiol.

[36] Griffiths PD, Coley SC, Romanowski CA, Hodgson T, Wilkinson ID (2003). Contrast-enhanced fluid-attenuated inversion recovery imaging for leptomeningeal disease in children. Am J Neuroradiol.

[37] Kan P, Liu JK, Hedlund G, Brockmeyer DL, Walker ML, Kestle JR (2006). The role of diffusion-weighted magnetic resonance imaging in pediatric brain tumors. Childs Nerv Syst.

[38] Sze G, Shin J, Krol G, Johnson C, Liu D, Deck MD (1988). Intraparenchymal brain metastases: MR imaging versus contrast-enhanced CT. Radiology.

[39] Sugahara T, Korogi Y, Tomiguchi S, Shigematsu Y, Ikushima I, Kira T, Liang L, Ushio Y, Takahashi M (2000). Posttherapeutic intraaxial brain tumor: the value of perfusion-sensitive contrast-enhanced MR imaging for differentiating tumor recurrence from nonneoplastic contrast-enhancing tissue. Am J Neuroradiol.

[40] Fleiss JL (1981). Statistical Methods for Rates and Proportion.

